# Sources of Potentially Toxic Elements in Sediments of the Mussulo Lagoon (Angola) and Implications for Human Health

**DOI:** 10.3390/ijerph17072466

**Published:** 2020-04-04

**Authors:** Pedro Dinis, Amílcar Armando, João Pratas

**Affiliations:** 1 MARE - Marine and Environmental Sciences Centre, Department of Earth Sciences, University of Coimbra, 3030-790 Coimbra, Portugal; 2 Universidade Metodista de Angola, Rua Nossa Senhora da Muxima 10, Caixa Postal 6739, Luanda, Angola; amilcarquizembe@hotmail.com; 3 Geosciences Center, Department of Earth Sciences, University of Coimbra, Rua Sílvio Lima, Univ. Coimbra – Pólo II, 3030-790 Coimbra, Portugal; jpratas@uc.pt

**Keywords:** Mussulo lagoon, Sediment composition, Factors controlling sediment geochemistry, Human Health Risk Assessment

## Abstract

The Mussulo lagoon is a coastal environment located near Luanda, one of the SW African cities that has been growing more rapidly during the last decades. Geochemical, mineralogical, and grain-size data obtained for the lagoon sediments are analyzed together, in order to establish the factors that control the distribution of some potentially toxic elements (PTEs). Sediments from northern location tend to be enriched in feldspar and, despite some variability in grain-size distributions, in fine-grained detrital minerals; southern lagoon sediments display very homogenous grain-size distribution and are enriched in minerals associated with salt precipitation (halite and gypsum). Multivariate statistics reveal a close link between some PTEs, namely Co, Hg, Ni, and Pb, for which an anthropogenic source can be postulated. On the other end, As seems to be associated with natural authigenic precipitation in southern lagoon sectors. Sediments enriched in clay also tend to yield more Fe, Mn, Zn, and Cu, but it is unclear whether their sources are natural or anthropogenic. Hazard indexes calculated for children are higher than 1 for As and Co, indicating potential non-carcinogenic risk. For the other elements, and for adults, there is no potential carcinogenic or non-carcinogenic risk.

## 1. Introduction

Luanda is one the African cities that has been growing more rapidly during the early 21st century [1]. The tendency for rapid rises in the number of inhabitants started before, in particular during the civil war after the independence of Angola, in 1975, when the population left the rural areas and sought refuge in the main cities. Hence, the city, projected to hold ~500,000 inhabitants, grew dramatically during the last decades, holding more than 10 times that number today. The rise in population also saw a rise in problems at the level of basic sanitation, collection, transport, and treatment of municipal solid waste, as well as limitations in the regulation of potentially hazardous waste disposal. Besides its large population, the city of Luanda also comprises the biggest industrial park in the country, leading to additional risks of environmental pollution. Some of the potentially hazardous wastes produced in Luanda are actually disposed of in open sites and dragged into the rivers and the sea during periods of rainfall. Thus, significant concentrations of potentially toxic elements (PTEs) are likely transported to coastal environments and can be concentrated in low hydrodynamic settings, such as the Mussulo lagoon, which is located a few km to the south of Luanda city centre.

Knowing that sediments in coastal ecosystems serve as sinks for PTEs, numerous investigations focused on the relations between the concentrations of PTEs in sediments and living organism have been conducted (e.g., [2,3,4,5]). High concentration of PTEs is frequently attributed to anthropogenic inputs [6,7,8,9,10,11], and strong relations between the levels of PTEs in coastal sediments and human activities were proposed for many locations worldwide [7,12,13,14,15,16,17,18]. Some works even established links between the history of human occupation and the concentration of harmful elements in coeval depositional sequences [13,14,16,18]. However, sediment geochemistry is necessarily controlled by the geology of the source-area. Furthermore, significant enrichments relative to source-rocks can be promoted by exogenous transformations due to weathering [19,20,21] and sorting processes [22,23,24]. Because of this complexity, understanding the factors responsible for the concentration of PTEs is of major importance in environmental studies.

In the present research, a set of textural, mineralogical, and geochemical properties of the sediments of the Mussulo lagoon are joint-analyzed, in order to investigate the factors that control the concentration of elements usually considered to be harmful in the environment. An assessment of the carcinogenic and non-carcinogenic risks associated with exposure to these sediments is also presented.

## 2. Geological and Geomorphological Setting

**Figure 1:**
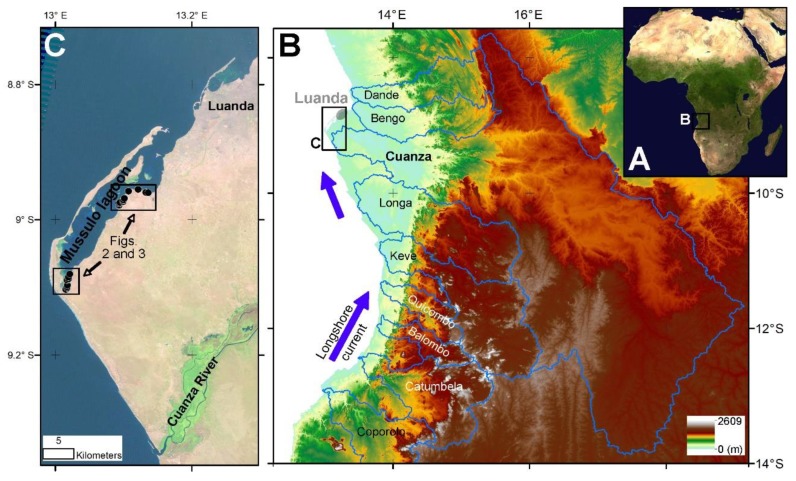
Geological setting of the lagoon of Mussulo. (**A**) The Angolan Atlantic margin in SW Africa and (**B**) orographic features of the regional drainage basins. Note that Cuanza is by far the biggest regional river. (**C**) The Mussulo spit and lagoon system extending until approximately the southern limit of Luanda urban area and location of the sampled sectors.

In central-west Angola, the Mussulo spit (~30 km long and <2 km in width) separates the elongated Mussulo lagoon from the South Atlantic (Figure 1). The spit is attached to the mainland some 30 km downdrift of the Cuanza River mouth, in a shifting point of coastal direction from SSE-NNW, southward, to SSW-NNE, northward. The lagoon reaches a maximum width of ~6 km in a bay near its aperture to the ocean. Approximately 6 km to the north of the tip of the Mussulo spit occur smaller linear features, including the so-called “Island of Luanda”. This was a narrow island (~12 km long and <500 in width) that, after human intervention in the first half of the 20th century and several reinforcements until present times, became permanently attached to the continent. 

Climatic conditions in coastal Angola are influenced by the cold, northward-flowing, Benguela Current that is responsible for the drier conditions than are seen at similar latitudes inland. In central and northern Angola, the climate evolves in ~200 km from semi-arid (Bsh type of Koppen) in the littoral to tropical savannah (Aw type of Koppen), a transition clearly evidenced by an increase in rainfall from less than 500 mm of annual precipitation to more than ~1000 mm. Further inland rainfall becomes even higher, reaching ~1500 mm in the most elevated areas draining towards the Atlantic Ocean. Annual average temperatures are usually above 25 °C near the coast, decreasing inland with an elevation of slightly less than 20°. In Luanda region, almost all rain occurs from October to April, with March and April being the months with the highest rainfall. The dry months tend to be slightly cooler, but still with average temperatures above 20°. The semi-arid conditions and the limited connectivity of the lagoon with the open ocean are responsible for high water salinity, averaging 39 in the dry season.

Throughout the Atlantic margin of Central Angola, the basement is made mostly of silica-rich plutonic and metamorphic rocks that belong to the Congo Craton, which also includes mafic complexes in its north-eastern tip [25,26]. To the north, the West Congo Supergroup, with a volcano-sedimentary succession, is covered by siliciclastic and carbonate strata outcrops along a large area that extends parallel to the coastline [27]. These basement units are overlain by Meso-Cenozoic successions deposited in the Benguela, Cuanza, and West Congo basins in association with the opening of the South Atlantic [28,29], and in the hinterland basins of Congo [30,31] and Kalahari [32]. Locally, thick tholeiitic rocks occur close to the contact between the basement and the sedimentary succession of the Cuanza Basin [33]. The natural development of the Mussulo spit and Luanda Island is strongly linked with persisting northward littoral transport, controlled by the oblique wave incidence on the coast. In Angola, where sand spits grow in the downdrift side of the mouth of major regional rivers, it is assumed that they are mostly sourced by bedload material supplied by these rivers [34,35]. But a comparison of the composition of sediments, collected at Cuanza River mouth with those from the Mussulo spit and the Island of Luanda, point to an additional sediment contribution that resembles the bedloads transported from the southern regions by coastal drift, or derived from volcanic rocks that outcrop in Mezo-Cenozoic basins [36]. 

At the time of its foundation, around 1575, Luanda was just a small colonial settlement that was designed for a limited number of families and was placed in “Luanda Island”. A year later, in search of better amenities, it moved to the mainland, and then evolved into the so-called village of “São Paulo de Loanda”. Most accurate estimations from the mid-18th century onward indicate that the population oscillated between a few thousand, and may have even decreased slightly during some periods [37], reaching more than 100,000 during the first half of the 20th century [38]. During the last decades of colonial occupation, the population started to grow more rapidly, becoming approximately 500,000 at the time of independence [39], ~3 million by the end of the 20th century, and ~6 million in 2018 [1]. Growth with limited land planning and the development of diverse industrial facilities likely promoted environmental pollution in potentially toxic metals. 

## 3. Materials and Methods

### 3.1. Sampling and Pre-Treatment

Samples of fine-grained sediments of the Mussulo bay were collected during October 2013 in areas exposed at low-tide periods. Sampling sites are clustered in outer (i.e., northern) and inner (i.e., southern) sectors of the Mussulo lagoon (Figure 1). All samples were air-dried in areas isolated of possible atmospheric contamination and sieved at 2 mm, before being analyzed in the laboratories of the Earth Sciences Department of University of Coimbra (DCT-UC). 

### 3.2. Analytical Procedures

The grain-size distributions of the sampled sediments were determined in the Sedimentology Laboratory of DCT-UC using a laser diffraction granulometer Coulter LS 230 that is able to measure the proportion of particles ranging from 0.5–2000 µm. Each sample was measured at least twice, and averaged results of these runs were used in the grain-size characterization.

The mineralogy was determined by X-ray diffraction (XRD) using a Philips^®®^ PW 3710 equipment with CuKα radiation and the software APD-PW1877 (version 3.6 J). Bulk mineralogy was determined on randomly oriented grains in the range 2–60° 2θ. Clay mineralogy was determined on oriented aggregates after pipetting clay suspension (<2 µm) to glass slides. XRD was then applied on air dried slides (2–30° 2θ) and after solvation with ethylene-glycol and heating at 550 °C (2–15° 2θ). Semi-quantitative estimations of mineral proportions are based on the areas of characteristic reflections after confirming the presence of the mineral with other XRD peaks. In bulk samples the following reflections were adopted: ~7.6 Å for gypsum, 4.26 Å for quartz, 3.23 Å for K-feldspar, 3.18 Å for plagioclase, 3.03 Å for calcite, 2.89 Å for dolomite, 2.82 Å for halite, and 2.72 Å for pyrite. In the clay fractions the glycolated diffractograms were adopted using the reflection of ~15–17 Å for smectite, ~12–13 Å for mixed layer clays, 10 Å for mica illite, 7.6 Å for gypsum, and 7.1 Å for kaolinite.

Concentrations of chemical elements were obtained with approximately 0.5 g of the fraction <0.18 mm of each sample. Dried sediment samples were placed in Teflon vessels (Multiwave 3000, Anton Paar) with 9 mL of 37% HCl, and 3 mL of 70% HNO_3_. The vessels were heated in a microwave apparatus within 10 min of ramp, and remained at about 180 °C for 15 min. The determination of total metal(loid) contents was performed using current analytical methods, including: Atomic Absorption Spectrometry (AAS, SOLAAR M Series equipment from Thermo Scientific, Madison, USA) for Ca, Cu, Co, Fe, Mg, Mn, Ni, and Zn with atomization source of flame. The same equipment using the grafite furnace mode was used to determine As, Co, and Pb, with ashing temperatures of 1100 °C, 1000 °C, and 800 °C, respectively; and atomization temperatures of 2600 °C, 2100 °C, and 1200 °C respectively. The observed detection limits were 0.05 mg/kg for As, Co and Hg, 0.3 mg/kg for Mg, 0.5 mg/kg for Pb and Zn, 1 mg/kg for Cu, and Ni, 3 mg/kg for Ca, Mn, and Fe. As a control, reference materials NIST 2709-San Joaquin Soil and RTC - CRM015 were used, and the recoveries obtained for the different elements showed values in the range of 86.2 and 100.7%, being highest for Fe and lowest for Ni. 

### 3.3. Statistical Data Treatment

Conventional univariate and multivariate statistical analysis were performed using the software JMP Pro 14.0. In order to better evaluate the associations between textural and compositional parameters obtained for the present investigation, a correlation-based Principal Component Analysis (PCA) was performed. A centered log–ratio transformation (clr) [40] was previously applied to the compositional data to remove the non-negativity and constant-sum constraints on compositional data. When the concentration of geochemical variables was below the detection limit in some samples, to allow their inclusion in the PCA, it adopted the square root of this limit divided by two. Supplementary Material ST1 shows the compositional data obtained for the present study.

### 3.4. Human Health Risk Assessment

The non-carcinogenic and carcinogenic risks were estimated according to the United States Environmental Protection Agency (USEPA) methodology [41]. The human health risk assessment was calculated assuming that both children and adult groups are directly exposed to potentially toxic elements (HMTE) hosted by sediments. Chronic Daily Intake (CDI.; mg·kg^−1^ bw per day) was determined for exposition to toxic elements by ingestion (CDI_ingest_), dermal contact (CDI_dermal_), and inhalation (CDI_inhalation_). The following equations were adopted:

(1) $${\mathrm{CDI}}_{\mathrm{ingest}}=\frac{C\times \mathrm{IR}\times \mathrm{EF}\times \mathrm{ED}\times \mathrm{CF}}{\mathrm{BW}\times \mathrm{AT}}$$

(2) $${\mathrm{CDI}}_{\mathrm{dermal}}=\frac{C\times \mathrm{SA}\times \mathrm{AF}\times \mathrm{ABS}\times \mathrm{EF}\times \mathrm{ED}\times \mathrm{CF}}{\mathrm{BW}\times \mathrm{AT}}$$

(3) $${\mathrm{CDI}}_{\mathrm{inhalation}}=\frac{C\times \mathrm{InhR}\times \mathrm{ET}\times \mathrm{EF}\times \mathrm{ED}}{\mathrm{PEF}\times \mathrm{BW}\times \mathrm{AT}}$$

where C is the concentration of PTEs in sediment (mg·kg^−1^). An explanation for the remaining parameters is presented in Table 1.

The human health non-carcinogenic risk caused by PTEs exposure is expressed as a hazard quotient (HQ) = CDI/RfD. The CDI is the average daily dose that a child or adult is exposed. The RfD is a reference dose, below which no adverse non-carcinogenic health effects should result from a lifetime of exposure. The HI is the chronic hazard index that is the sum of the hazard quotients for multiple exposure pathways. For HI values > 1, there is a chance that non-carcinogenic risk may occur; otherwise, the individuals are exposed to concentrations that do not present a hazard. The carcinogenic risks (CR) for As and Ni exposure of the studied groups were calculated according to the Exposure Factors Handbook [41] and using the Slope Factors according to the U.S. Department of Energy (USDE) [42]. 

## 4. Results and Discussion

### 4.1. Compositional Variability Within the Lagoon

#### 4.1.1. Grain-Size

**Figure 2:**
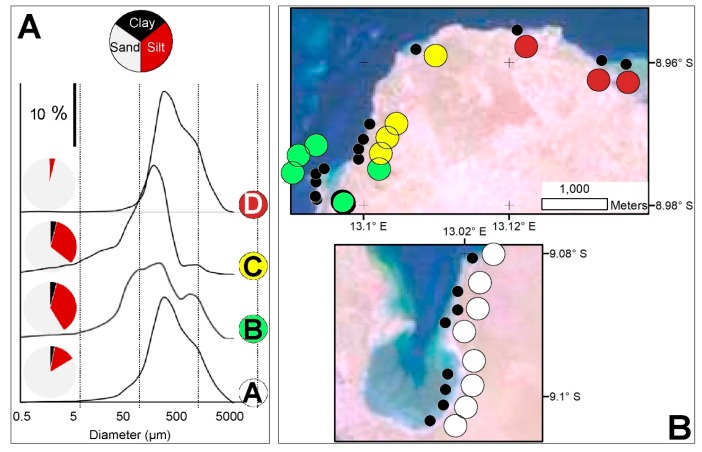
Grain-size of the Mussulo lagoon sediments. General features of different grain-size types (**A**) and their spatial distribution (**B**).

The sampled sediments can be organized into four groups based on their grain-size distributions (Figure 2). Samples collected in inner lagoon locations (group A.; samples A1 to A8) are characterized by a clear predominance of sand-size particles (95–98%) and very low clay content (<1%), displaying coarse-skewed unimodal distributions with modal sizes in classes ranging 0.12–0.25 mm. In western locations of the northern sectors of the lagoon, finer-grained sediments (58% < sand < 77%) predominate, which tend to show relatively wide grain-size distributions, frequently with a main mode in the interval 63–177 µm and a secondary population with modal size in the interval 0.25–0.5 mm (group B.; samples A9 to A15). Sediments further north (group C.; samples A16 to A19) are characterized by fine-skewed unimodal distributions, which tend to become finer northwards with decreasing sand content (from 70% to 40%), increasing silt (from 27% to 51%) and clay (from 3% to 6%) content, and a modal-size evolving from 0.088–0.125 to 0.063–0.088 mm. Finally, in the outermost locations of the lagoon (group D.; samples A20 to A22), grain-size becomes coarser again, with higher sand content (79%–89%), but still with significant amounts of clay (up to 5%).

#### 4.1.2. Mineralogy

**Figure 3:**
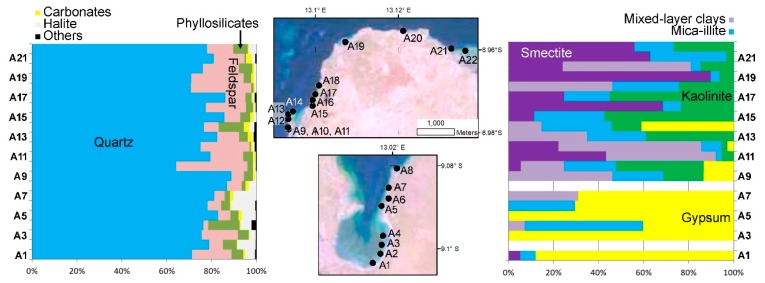
Bulk and clay mineral assemblages obtained for sediments of the Mussolo lagoon.

Sediment mineralogy for bulk samples and the clay fraction are represented in Figure 3. The sediments of Mussolo lagoon are strongly enriched in quartz (65%–89%), followed by feldspars (2%–32%), phyllosilicates (1%–14%), and halite (<1%–13%). Gypsum, calcite, and dolomite, are usually present, but always in minor or trace amounts (<3%). Traces of pyrite are occasionally found and Mg-salts (e.g., carnalite, kainite, polyhalite) may also be present. Halite contents are higher in southern (3%–13%) than in northern locations (1%–4%), whilst feldspar tends to display an opposite spatial distribution (Figure 3). There are no clear geographic trends for quartz content or the remaining minerals.

The composition of the clay fraction is highly variable and can be organized in three mineral assemblages with specific spatial distribution. Most sediments collected in southern realms of the lagoon (samples A1, A3, A5, and A6) are strongly enriched in gypsum (40%–100%), followed by mica-illite (0%–30%). A second assemblage is characterized by an enrichment in expansive clays (smectite and mixed-layer clays; 72%–100%), usually with secondary amounts of mica-illite (6%–26%) and kaolinite (3%–21%). This clay-mineral assemblage is characteristic of samples collected near the mouth of coastal streams in the northern sector of the lagoon (samples A11, A12, A20, and A21). Sediments from the northern sector of the lagoon can also yield a mix composition, with a variable abundance of kaolinite (13%–59%) and expansive clays (<55%), and more homogenous mica-illite (13%–25%).

The opposite behavior of feldspar and halite can be regarded as evidence of different orthochemical and detrital contributions. Because of the semi-arid climatic conditions and the high-water salinities, chemical precipitation in inner lagoon sectors likely occurs. The overall higher gypsum content in the clay fraction of the samples collected in the south can also be ascribed to authigenic formation in saline environments. Higher detrital supply, either with marine or continental sources, should occur in the northern part of the lagoon.

#### 4.1.3. Geochemistry

**Figure 4:**
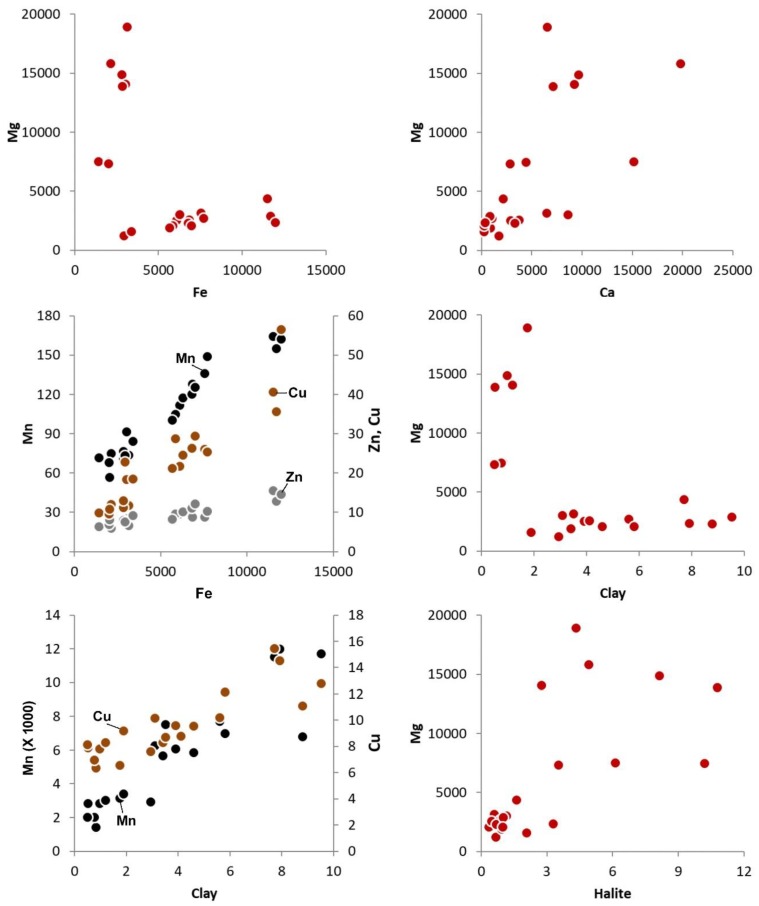
Ratios between chemical element concentrations and mineralogical and textural features of the sampled sediments. The plots Fe vs. Mg and clay (%) vs. Mg show two groups of samples, both displaying trends for increasing Mg with Fe and clay. Although with high scattering, Mg appears to correlate with Ca and halite. Samples enriched in clay also tend to yield higher Cu, Mn, Zn, and Fe.

Eleven chemical elements were selected for this research (As, Ca, Cu, Co, Fe, Hg, Mg, Mn, Ni, Pb, and Zn). As expected for sediments of a lagoon environment with high salinity, Ca (0.03%–1.98%) and Mg (0.12%–1.89%) are among the elements with highest measured concentration, and they tend to be more abundant in inner lagoon locations. Iron is more evenly distributed throughout the Mussulo lagoon (0.14%–1.20%), although, in general, with higher contents in northern samples. Samples enriched/depleted in Fe also yield high/low Cu (5.97–15.47 mg/kg), Mn (56.71–164.28 mg/kg) and Zn (9.59–56.48 mg/kg) contents (Figure 4).

Arsenic and Co were found in all samples, but in wide variable abundances (0.09–31.49 mg/kg and 0.78–26.04 mg/kg, respectively) and high contents can occur both in northern and southern locations. The other measured elements (Hg, Ni, and Pb) were detected only in part of the samples, Hg in eight of them (<0.12 mg/kg), Ni in 11 (<13.34 mg/kg), and Pb in 12 (<30.12 mg/kg). Sediments with relatively high amounts of at least two of these elements were collected both in the northern sector of the lagoon, namely near Luanda (samples A20, A21, and A22), and in some of the innermost southern locations (samples A2, A3, A6, and A8).

The plots of Mg vs. Fe and of Mg vs. grain-size variables reveal two distinct groups (Figure 4). Finer samples collected in the northern sector yield lower Mg and higher Fe; coarser samples collected in the southern sector yield higher Mg and lower Fe. However, if the two groups are isolated, Mg concentrations appear to be higher in finer sediments, which also tend to be enriched in Fe. The trend for higher Mg in sediments enriched in halite supports the possibility that Mg is strongly influenced by authigenic mineral formation, which prevails in southern lagoon settings with coarser sediments. On the other hand, the distribution of the two groups of samples in the bi-plots suggests that the presence of detrital fine-grained particles hosting Mg and Fe, along with other siderophile elements, is also influencing their concentrations.

### 4.2. Factors Controlling Sediment Composition

**Figure 5:**
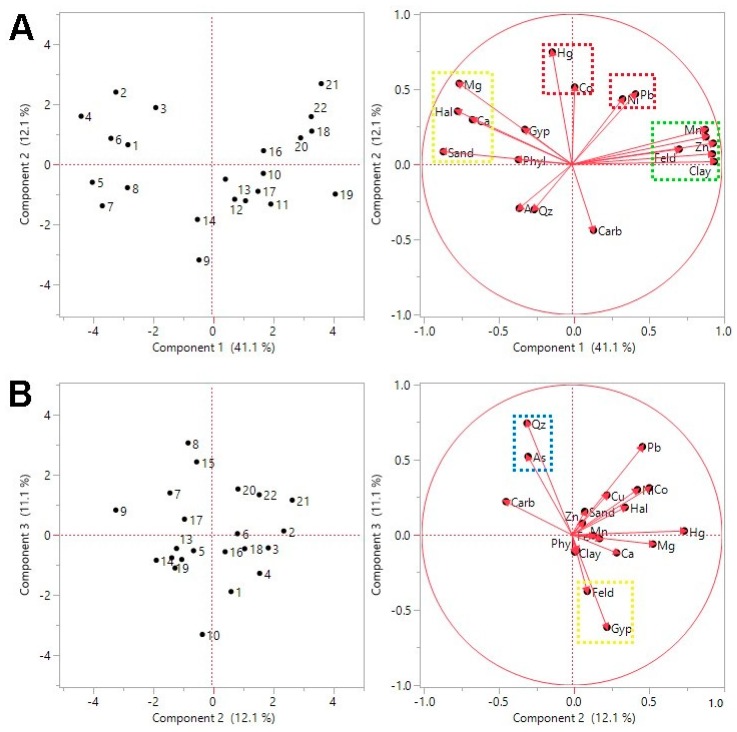
Maps of the principal components for geochemical, grain-size and mineralogical variables. (**A**) The vector loadings for the two main components of the Principal Component Analysis (PCA) define two perpendicular links, indicating two independent controls on the data. The first link connects elements assumed to be carried with fine-grained detrital minerals in opposition to elements that may result from precipitation in the lagoon. The second link connects a set of elements that may have anthropogenic sources. (**B**) The plot of PC2 vs. PC3 shows the opposition of quartz and feldspar, two detrital minerals whose relative enrichment in the lagoon sediments can be attributed to specific source areas. Gypsum seems to be associated with feldspar, which is compatible with a source in Meso-Cenozoic units of the neighboring Cuanza Basin.

A PCA using grain-size, bulk mineralogy, and geochemical variables helps to ascertain the compositional variability for the studied sediments. Variables strongly correlated or anti-correlated were not considered in the PCA. Clay mineralogy was also excluded because of the low amount of clay fraction in the studied sediments, such that in two samples it was not possible to establish clay assemblages, and the fact that no mineral was detected in all samples. The first three components explain 64.3% of the observed variance (Figure 5).

The first component (PC1, 41.7% of the variance) reflects a contrast between a set of variables that are probably linked with chemical or biochemical precipitation, such as halite, Mg, and Ca, and others linked with detrital supply, such as feldspar, Fe, Mn, Zn, and Cu. It also separates coarser sediments with high negative loadings of precipitation-associated variables, from finer sediments with high positive loadings of detrital-associated variables, reinforcing the possibility that a significant proportion of the coarser particles are authigenic and not physically transported to the lagoon (Figure 5). Based on the relations between geochemical and grain-size variables, it can be assumed that most Zn and Cu are hosted by fine-grained particles, although one cannot draw conclusions about whether they have natural or anthropogenic sources. It is interesting to note that Ca and Mg are not correlated with carbonate content, indicating that other minerals are hosting these elements. The correlation between halite and Mg suggest that Mg-bearing salts are being precipitated in the lagoon, as suggested before. XRD data is compatible with the possible occurrence of traces of Mg-salts. 

Relatively low loadings of PC1 were also obtained for As, associating this element with the authighenic variables. Arsenic concentrations in non-contaminated near shore or estuarine sediments are in the order of 5–15 mg/kg [43], which encompasses the majority of the values obtained for Mussulo sediments. This element tends to be enriched in sea water relative to river water [44] and it can precipitate in reduced marine environments [45]. Taking into consideration the influence of authigenic salt-minerals on sediment composition and the occurrence of traces of pyrite in some samples, it is probable that the relatively high As concentration in some inner lagoon locations is related with local salt-mineral formation.

The second (PC2) and third (PC3) components explain substantially lower proportions of total variability (13.1% and 10.2%, respectively). PC2 yields high positive loadings of Hg, Pb, Ni, and Co, all elements for which a source related to human activities can be postulated [7,8,9,10,11,13,14,15,16,17,18]. The plot PC1 vs. PC2 (Figure 5) shows the links among this group of elements, suggesting a closer association of Pb and Ni with the variables with high loadings of PC1 (clay, Fe, Mn, Zn, and Cu), whilst Co is largely independent of PC1. Arsenic is not plotted with these elements, reinforcing the possibility of a natural origin associated with salt precipitation in the lagoon. Samples with high scores of this component are from both the northern and southern sectors.

PC3 shows an opposition between quartz and feldspar (Figure 5), which are the two most common minerals in the studied sediments, being both of detrital origin. Gypsum appears linked with feldspar. Differences between the sands of the Mussulo spit and at the mouth of the Cuanza river, which yield less feldspar along with alkali and alkaline earth metals, and more quartz and ultra-stable heavy minerals [36], support the possibility of different detrital sources in this costal environment. We propose here that sediment material enriched in feldspar and gypsum came from a proximal source, most likely the previous-cycle depositional units of the Cuanza Meso-Cenozoic basin that are enriched in these minerals; quartz-rich sediment is probably derived from more distant regions (i.e., the Cuanza River mouth and further up-drift). Except for As, which displays a close link with quartz, this component does not seem to have a major influence on the distribution of the studied PTEs.

In summary, an anthropogenic influence on sediment composition is probably responsible for occasional enrichments in Hg, Pb, Ni, and Co. Other elements found in higher abundances in fine grained sediments near Luanda, such as Cu and Zn, may also be partially human derived. On the other hand, natural factors seem to account for the observed distribution of As contents.

### 4.3. Health Implications of Sediment Composition

High concentrations of PTEs in near-surface environment can threaten human health via sediment ingestion (geophagism), rare in adults but quite common in children, or by hand to-mouth intake, inhalation of dust particles, or dermal contact [46,47]. The Hazard Indices obtained from the health risk assessment for Cu, Hg, Mn, Ni, Pb, and Zn were below 1, pointing to no non-carcinogenic risk (Table 2). Regarding As and Co for children, HI reached 1.2 and 1.1, respectively, indicating potential non-carcinogenic risk due to sediment exposure. The proportion of cases with HI > 1 is 5% for As and 9% for Co. According to these results, it is highly recommendable that children spent less time playing in the lagoon sediments and avoid hand-to-mouth intake.

According to the International Agency on Research of Cancer (IARC) [48], of the analyzed elements, only As and Ni poses significant carcinogenic risk. Cancer Risks values determined with the sampled sediments of the Mussulo lagoon (Table 2) are within the classes of acceptable carcinogenic risk for As (1 × 10^−4^ to 1 × 10^−6^; [41]) and no risk for Ni (<1 × 10^−6^; [27]).

## 5. Conclusions

The geochemical composition of the sediments of the Mussulo lagoon is mainly determined by natural processes that influence the abundance of detrital components, which can be derived from different source areas, and authigenic components associated with mineral precipitation within the lagoon setting and surrounding regions. Human action may have a subsidiary role, contributing to the abundance of some PTEs, such as Co, Hg, Ni, and Pb, in parts of the lagoon sediments. Fine grained samples collected near Luanda yield relatively high concentrations of Zn and Cu, but whether their sources are natural or anthropogenic is uncertain. Arsenic is more abundant in inner (i.e., southern) locations of the lagoon, probably in association with natural processes due to precipitation from salt-water. The health risk assessments suggest no major carcinogenic risk due to sediment intake, but the concentrations of As and Co indicate potential non-carcinogenic risk for children.

The present research shows that health risks associated with element concentrations can emerge from enrichment due to both natural and anthropogenic processes. Although some PTEs preferentially hosted by fine grained particles (e.g., Co, Hg, Ni, Pb, Zn, and Cu) may be associated with human activities, in coastal settings of arid regions, the possibility of high concentrations of As due to natural precipitation should be fully considered in environmental assessments.

## Figures and Tables

**Table 1:** Values adopted for the parameters used in the determination of Chronic Daily Intake (CDI) based on USEPA (2011).

Parameter	Adult	Children
IR (Ingestion Rate of sediment)	100 mg·day^−1^	200 mg·day^−1^
EF (Exposure Frequency)	312 days·year^−1^	312 days·year^−1^
ED (Exposure Duration)	35 years	6 years
BW (Body Weight)	15 kg	70 kg
AT (Averaging Time for non-carcinogenic risk	365 days × 6	365 days × 35
AT (Averaging Time for carcinogenic risk	365 days × 70	365 days × 70
CF (Conversing Factor)	10–6 mg·day^−1^	10–6 mg·day^−1^
SA (Skin Surface Area available for contact)	6032 cm^2^	2373 cm^2^
AF (Soil to skin Adherence Factor)	0.07 mg cm^−2^	0.2 mg cm^−2^
ABS (Absorption Factor)	0.001	0.001
InhR (Inhalation Rate)	1.56 m^3^·h^−1^	1.2 m^3^·h^−1^
ET (Exposure Time)	8 h·day^−1^	4 h·day^−1^
PEF (Particle Emission Factor)	1.36 × 109 m^3^·kg^−1^	1.36 × 109 m^3^·kg^−1^

**Table 2:** Range of results obtained for Hazard Indexes (HI) and Cancer Risks (CR) due to sediment exposure for the different PTEs. Maximum results that are above the target values are in bold.

	HI		CR	
	Children	Adult	Children	Adult
As	0.0–**1.2**	0.0–0.1	9 × 10^−10^ to 5 × 10^−5^	3 × 10^−11^ to 3 × 10^−5^
Mn	0.0–0.2	0.0–0.0		
Co	0.0–**1.1**	0.0–0.1		
Cu	0.0–0.0	0.0–0.0		
Hg	0.0–0.1	0.0–0.0		
Ni	0.0–0.0	0.0–0.0	1 × 10^−11^ to 1 × 10^−9^	4 × 10^−11^ to 1 × 10^−10^
Pb	0.0–0.1	0.0–0.0		
Zn	0.0–0.0	0.0–0.0		

## Supplementary Materials

The following are available online at https://www.mdpi.com/1660-4601/17/7/2466/s1.

## References

[1] UN (United Nations) (2018). The World’s Cities in 2018: Data Booklet, Statistical Papers—United Nations (Ser. A), Population and Vital Statistics Report, UN, New York.

[2] Cheggour M., Chafik A., Langston W.J., Burt G.R., Benbrahim S., Texier H. (2001). Metals in sediments and the edible cockle Cerastoderma edule from two Moroccan Atlantic lagoons: Moulay Bou Selham and Sidi Moussa. Environ. Pollut..

[3] Fernandes C., Fontainhas-Fernandes A., Peixoto F., Salgado M.A. (2007). Bioaccumulation of heavy metals in Liza saliens from the Esmoriz–Paramos coastal lagoon, Portugal. Ecotoxicol. Environ. Saf..

[4] Spada L., Annicchiarico C., Cardellicchio N., Giandomenico S., Di Leo A. (2012). Mercury and methylmercury concentrations in Mediterranean seafood and surface sediments, intake evaluation and risk for consumers. Int. J. Hyg. Environ. Health.

[5] María-Cervantes A., Jiménez-Cárceles F.J., Álvarez-Rogel J. (2009). As, Cd, Cu, Mn, Pb, and Zn contents in sediments and mollusks (Hexaplex trunculus and Tapes decussatus) from coastal zones of a Mediterranean lagoon (Mar Menor, SE Spain) affected by mining wastes. Water Air Soil Pollut..

[6] Pinto M.M., Silva M.M., Neiva A.M. (2004). Pollution of water and stream sediments associated with the Vale De Abrutiga Uranium Mine, Central Portugal. Mine Water Environ..

[7] Pekey H. (2006). The distribution and sources of heavy metals in Izmit Bay surface sediments affected by a polluted stream. Mar. Pollut. Bull..

[8] Biasioli M., Grčman H., Kralj T., Madrid F., Díaz-Barrientos E., Ajmone-Marsan F. (2007). Potentially toxic elements contamination in urban soils. J. Environ. Qual..

[9] Qingjie G., Jun D., Yunchuan X., Qingfei W., Liqiang Y. (2008). Calculating pollution indices by heavy metals in ecological geochemistry assessment and a case study in parks of Beijing. J. China Univ. Geosci..

[10] Ye S., Zeng G., Wu H., Zhang C., Liang J., Dai J., Liu Z., Xiong W., Wan J., Xu P. (2017). Co-occurrence and interactions of pollutants, and their impacts on soil remediation—A review. Crit. Rev. Environ. Sci. Technol..

[11] Cabral-Pinto M.M., Inácio M., Neves O., Almeida A.A., Pinto E., Oliveiros B., da Silva E.A. (2019). Human health risk assessment due to agricultural activities and crop consumption in the surroundings of an industrial area. Expo. Health.

[12] Mirlean N., Andrus V.E., Baisch P., Griep G., Casartelli M.R. (2003). Arsenic pollution in Patos Lagoon estuarine sediments, Brazil. Mar. Pollut. Bull..

[13] Elbaz-Poulichet F., Dezileau L., Freydier R., Cossa D., Sabatier P. (2011). A 3500-year record of Hg and Pb contamination in a Mediterranean sedimentary archive (The Pierre Blanche Lagoon, France). Environ. Sci. Technol..

[14] Covelli S., Langone L., Acquavita A., Piani R., Emili A. (2012). Historical flux of mercury associated with mining and industrial sources in the Marano and Grado Lagoon (northern Adriatic Sea). Estuar. Coast. Shelf Sci..

[15] Fujita M., Ide Y., Sato D., Kench P.S., Kuwahara Y., Yokoki H., Kayanne H. (2014). Heavy metal contamination of coastal lagoon sediments: Fongafale Islet, Funafuti Atoll, Tuvalu. Chemosphere.

[16] Alyazichi Y.M., Jones B.G., McLean E. (2015). Source identification and assessment of sediment contamination of trace metals in Kogarah Bay, NSW, Australia. Environ. Monit. Assess..

[17] Ke X., Gui S., Huang H., Zhang H., Wang C., Guo W. (2017). Ecological risk assessment and source identification for heavy metals in surface sediment from the Liaohe River protected area, China. Chemosphere.

[18] Mejjad N., Laissaoui A., El-Hammoumi O., Fekri A., Amsil H., El-Yahyaoui A., Benkdad A. (2018). Geochemical, radiometric, and environmental approaches for the assessment of the intensity and chronology of metal contamination in the sediment cores from Oualidia lagoon (Morocco). Environ. Sci. Pollut. Res..

[19] Lottermoser B.G. (1997). Natural enrichment of topsoils with chromium and other heavy metals, Port Macquarie, New South Wales, Australia. Soil Res..

[20] Kraepiel A.M., Dere A.L., Herndon E.M., Brantley S.L. (2015). Natural and anthropogenic processes contributing to metal enrichment in surface soils of central Pennsylvania. Biogeochemistry.

[21] Pinto M.M., Silva M.M., da Silva E.A., Dinis P.A., Rocha F. (2017). Transfer processes of potentially toxic elements (PTE) from rocks to soils and the origin of PTE in soils: A case study on the island of Santiago (Cape Verde). J. Geochem. Explor..

[22] Komar P.D., Mange M.A., Wright D.T. (2007). The entrainment, transport and sorting of heavy minerals by waves and currents. Heavy Minerals in Use.

[23] Garzanti E., Andò S., Vezzoli G. (2009). Grain-size dependence of sediment composition and environmental bias in provenance studies. Earth Planet. Sci. Lett..

[24] Garzanti E., Dinis P., Vermeesch P., Andò S., Hahn A., Huvi J., Limonta M., Padoan M., Resentini A., Rittner M. (2018). Sedimentary processes controlling ultralong cells of littoral transport: Placer formation and termination of the Orange sand highway in southern Angola. Sedimentology.

[25] De Carvalho H., Tassinari C., Alves P.H., Guimarães F., Simões M.C. (2000). Geochronological review of the Precambrian in western Angola: Links with Brazil. J. Afr. Earth Sci..

[26] Vaughan A.P., Pankhurst R.J. (2008). Tectonic overview of the West Gondwana margin. Gondwana Res..

[27] Tack L., Wingate M.T., Liégeois J.P., Fernandez-Alonso M., Deblond A. (2001). Early Neoproterozoic magmatism (1000–910 Ma) of the Zadinian and Mayumbian Groups (Bas-Congo): Onset of Rodinia rifting at the western edge of the Congo craton. Precambrian Res..

[28] Moulin M., Aslanian D., Unternehr P. (2010). A new starting point for the South and Equatorial Atlantic Ocean. Earth Sci. Rev..

[29] Guiraud M., Buta-Neto A., Quesne D. (2010). Segmentation and differential post-rift uplift at the Angola margin as recorded by the transform-rifted Benguela and oblique-to-orthogonal-rifted Kwanza basins. Mar. Pet. Geol..

[30] Daly M.C., Lawrence S.R., Diemu-Tshiband K., Matouana B. (1992). Tectonic evolution of the Cuvette Centrale, Zaire. J. Geol. Soc..

[31] Kadima E., Delvaux D., Sebagenzi S.N., Tack L., Kabeya S.M. (2011). Structure and geological history of the Congo Basin: An integrated interpretation of gravity, magnetic and reflection seismic data. Basin Res..

[32] Haddon I.G., McCarthy T.S. (2005). The Mesozoic–Cenozoic interior sag basins of Central Africa: The late-cretaceous–Cenozoic Kalahari and Okavango basins. J. Afr. Earth Sci..

[33] Marzoli A., Melluso L., Morra V., Renne P.R., Sgrosso I., D’antonio M., Morais L.D., Morais E.A., Ricci G. (1999). Geochronology and petrology of Cretaceous basaltic magmatism in the Kwanza basin (western Angola), and relationships with the Paraná-Etendeka continental flood basalt province. J. Geodyn..

[34] Abecasis C.K. (1961). As Formações Lagunares e Seus Problemas de Engenharia Litoral.

[35] Dinis P.A., Huvi J., Cascalho J., Garzanti E., Vermeesch P., Callapez P. (2016). Sand-spits systems from Benguela region (SW Angola). An analysis of sediment sources and dispersal from textural and compositional data. J. Afr. Earth Sci..

[36] Garzanti E., Dinis P., Vermeesch P., Andò S., Hahn A., Huvi J., Limonta M., Padoan M., Resentini A., Rittner M. (2018). Dynamic uplift, recycling, and climate control on the petrology of passive-margin sand (Angola). Sediment. Geol..

[37] Curto J.C., Gervais R.R. (2002). A dinâmica demográfica de Luanda no contexto do tráfico de escravos do Atlântico Sul, 1781–1844. Topoi (Rio de Janeiro).

[38] Amaral I.D. (1959). Subsídios para o estudo da evolução da população de Luanda. Garcia Orta Rev. Junta Missões Geográficas Investig. Do Ultramar.

[39] Amaral I. (1983). Luanda e os seus “muceques”, problemas de Geografia Urbana. Finisterra.

[40] Aitchison J. (1982). The statistical analysis of compositional data. J. R. Stat. Soc. Ser. B (Methodological).

[41] USEPA (United States Environmental Protection Agency) (2011). Exposure Factors Handbook 2011 Edition (Final).

[42] USDE (U.S. Department of Energy) (2013). The Risk Assessment Information System (RAIS).

[43] Neff J.M. (1997). Ecotoxicology of arsenic in the marine environment. Environ. Toxicol. Chem. Int. J..

[44] Dong W.Q., Cui Y., Liu X. (2001). Instances of soil and crop heavy metal contamination in China. Soil Sediment Contam..

[45] Kalia K., Khambholja D.B., Flora S.J.S. (2015). Arsenic contents and its biotransformation in the marine environment. Handbook of Arsenic Toxicology.

[46] Viers J., Dupré B., Gaillardet J. (2009). Chemical composition of suspended sediments in World Rivers: New insights from a new database. Sci. Total Environ..

[47] Sun Y., Zhou Q., Xie X., Liu R. (2010). Spatial, sources and risk assessment of heavy metal contamination of urban soils in typical regions of Shenyang, China. J. Hazard. Mater..

[48] IARC (International Agency for Research on Cancer) (2017). List of Classifications 1–123. https://monographs.iarc.fr/agents-classified-by-the-iarc/.

