# Supplementary material for: Sources of Potentially Toxic Elements in Sediments of the Mussulo Lagoon (Angola) and Implications for Human Health
Source: Int J Environ Res Public Health. 2020 Apr 4;17(7):2466. doi: 10.3390/ijerph17072466 (PMC7177521; doi:10.3390/ijerph17072466)
Supplement: Supplementary file 1 [file ijerph-17-02466-s001.pdf]

	Geochemistry											Grain-size			Mineralogy					
	Ca	Co	Cu	Fe	Mg	Mn	Zn	As	Ni	Pb	Hg	Clay (<4µ	Silt (4-63	Sand (>6	Gypsum	Quartz	Feldspar	Phyllosili	Carbonat	Halite
	mg/kg	mg/kg	mg/kg	mg/kg	mg/kg	mg/kg	mg/kg	mg/kg	mg/kg	mg/kg	µg/kg	%	%	%	%	%	%	%	%	%
A1	6557	1.60	6.56	3137	18935	73.8	11.81	7.83	<1	<0.5	196.47	1.75	13.24	85.01	1	71	18	5	1	4
A2	9634	4.97	7.80	2826	14896	70.9	11.15	1.92	11.07	1.88	157.09	0.98	7.08	91.94	0	79	6	6	0	8
A3	9258	8.34	8.31	3030	14045	91.5	18.34	9.19	8.75	<0.5	112.48	1.19	9.93	88.88	0	76	16	5	0	3
A4	19824	17.29	5.97	2138	15840	75.0	11.97	0.64	<1	<0.5	96.22	1.20	8.80	90.00	2	76	2	14	1	5
A5	15172	1.38	6.35	1422	7502	71.5	9.90	0.85	<1	<0.5	70.07	0.82	6.41	92.76	0	83	6	4	2	6
A6	7103	9.53	7.91	2840	13896	76.6	12.95	2.43	<1	2.78	<50	0.52	3.10	96.38	1	78	6	4	1	11
A7	4403	7.59	6.96	2005	7444	67.8	9.59	17.06	<1	<0.5	<50	0.76	4.52	94.72	0	81	5	2	2	10
A8	2852	6.57	8.13	2026	7328	56.7	10.82	31.49	2.37	18.05	<50	0.50	2.71	96.79	0	87	7	2	1	4
A9	1713	1.17	7.63	2932	1216	73.4	22.83	5.66	<1	<0.5	<50	2.94	34.32	62.74	0	89	6	4	1	1
A10	2908	2.82	9.60	6088	2512	111.5	21.79	0.25	<1	<0.5	<50	3.90	43.14	52.95	0	64	32	3	1	1
A11	534	1.30	9.57	5865	2070	104.8	28.67	0.98	13.34	<0.5	<50	4.60	40.04	55.37	0	80	15	4	1	0
A12	905	2.67	8.28	5653	1901	100.4	21.17	2.96	<1	1.30	<50	3.41	29.66	66.93	0	75	22	1	1	1
A13	6493	7.94	8.72	7534	3136	136.1	26.08	1.33	<1	<0.5	<50	3.51	31.46	65.03	0	82	10	4	2	1
A14	250	4.68	9.19	3388	1579	84.2	18.42	0.47	<1	<0.5	<50	1.90	15.23	82.87	1	77	7	11	3	2
A15	8596	15.32	10.16	6276	3007	117.5	24.56	16.65	3.83	3.11	<50	3.09	20.92	75.98	0	86	7	4	2	1
A16	3748	24.00	8.78	6831	2570	127.8	26.51	0.82	<1	11.08	<50	4.10	44.24	51.65	0	77	18	3	1	0
A17	1049	5.97	10.22	7712	2704	148.8	25.38	14.53	<1	1.42	<50	5.61	45.79	48.60	0	86	9	3	1	1
A18	3299	26.04	11.07	6792	2311	120.2	26.24	0.09	2.90	9.56	<50	8.79	47.46	43.75	0	71	27	1	1	1
A19	827	0.78	12.78	11697	2883	154.8	35.58	2.91	6.04	0.51	<50	9.52	63.86	26.62	0	71	23	3	1	1
A20	259	8.92	12.15	6977	2087	125.3	29.47	7.55	4.36	30.12	96.04	5.81	50.40	43.79	0	76	16	6	1	1
A21	2166	11.12	15.47	11503	4344	164.3	40.58	0.30	12.53	19.72	1017.75	7.72	43.89	48.40	0	81	14	1	3	2
A22	344	6.56	14.52	11982	2358	162.4	56.48	0.53	1.40	26.52	89.32	7.92	16.73	75.35	0	78	12	6	1	3
